# The efficacy of four-slice helical CT in evaluating pancreatic trauma: a single institution experience

**DOI:** 10.1186/1752-2897-5-1

**Published:** 2011-01-07

**Authors:** Wei-Jing Lee, Ning-Ping Foo, Hung-Jung Lin, Yen-Chang Huang, Kuo-Tai Chen

**Affiliations:** 1 Emergency Department, Chi-Mei Medical Center, 901 Chung-Hwa Road, Yung Kang, Tainan 710, Taiwan ROC

## Abstract

**Study objective:**

To assess the efficacy of computed tomography (CT) in evaluating patients with pancreatic trauma.

**Methods:**

We undertook a retrospective review of all blunt trauma patients admitted to the Chi-Mei Medical Center from January 2004 to June 2006. Every patients underwent abdominal CT scan in emergency department and the CT scans were obtained with a four-slice helical CT. Diagnosis of a pancreatic injury in these patients was by surgical observation or by CT findings. Radiographic pancreatic injuries were classified as deep or superficial lesions. Deep lesions were defined as the hematomas or lacerations >50% thickness of the pancreas. Superficial lesions were described as the hematomas or lacerations <50% thickness of the pancreas; pancreatic edema; and focal fluid accumulation around the pancreas

**Results:**

Nineteen patients with pancreatic trauma, fourteen males and five females, average age 40.6 ± 21.4 years, were included. Most patients (73.7%) with pancreatic trauma had associated organ injuries. CT was performed in all patients and laparotomy in 14 patients. CT was 78.9% sensitive in detecting pancreatic trauma. All deep pancreatic lesions revealed on CT required surgical treatment, and complication was discovered in two patients undergoing delayed surgery. Superficial lesions were managed conservatively.

**Conclusion:**

Four-slice helical CT can detect most pancreatic trauma and provide practical therapeutic guidance. Delayed operation might result in complications and is associated with prolonged hospital stays.

## Introduction

Pancreatic trauma is uncommon and most trauma surgeons have little experience in managing the condition [1,2]. To complicated matters, most patients with pancreatic trauma have concomitant injuries, [2] which frequently obscure the symptoms of pancreatic trauma and distract the attention of the trauma surgeon. Serum amylase and lipase tests have been proved neither sensitive nor specific [3,4]. Moreover, the pancreas is deeply seated in the retroperitoneum and there are difficulties using physical examination, sonography and diagnostic peritoneal lavage to investigate this area [1,5,6].

Torso computed tomography scanning (CT) is currently the most useful tool in evaluating patients who have sustained torso trauma. The majority of stable trauma patients with a high suspicion of intra-abdominal organ injuries require CT imaging. However, the reliability of CT in detecting pancreatic trauma is still debated [7-9].

The aim of this study is to assess the efficacy of CT in evaluating patients with pancreatic trauma and examine how CT findings influence the management of these patients.

## Methods

This study was approved by the Institutional Review Board of Chi-Mei Medical Center. We performed a retrospective chart review in all blunt trauma patients admitted to Chi-Mei Medical Center from July 2003 to September 2006. Papers and electronic medical records were searched by the five authors to identify the cases of pancreatic injury. Diagnosis of a pancreatic injury in these patients was by surgical observation (14 patients) or by CT findings (5 patients). Every patients underwent abdominal CT scan in emergency department and the CT scans were obtained with a helical CT(Four Slice: HiSpeed CT, GE). All patients received intravenous contrast material and the image thicknesses are 5 or 7.5 mm. Data reviewed contained demographic information, mechanisms of trauma, Injury Severity Score (ISS), CT findings, associated organ injuries, operative results, time to operation, length of hospital and intensive care unit (ICU) stay, complications and mortality.

According to the CT images, radiographic pancreatic injuries were classified as deep or superficial lesions. Deep lesions were defined as the hematomas or lacerations >50% thickness of the pancreas. Superficial lesions were described as the hematomas or lacerations <50% thickness of the pancreas; pancreatic edema; and focal fluid accumulation around the pancreas [10,11]. The American Association for the Surgery of Trauma Organ Injury Score for pancreatic injury was not used in this study, because only few surgical or radiological reports remarked the integrity of pancreatic duct (Table 1).

**Table 1:** American Association for the Surgery of Trauma Organ Injury Score for pancreatic injury

Grade	Injury	Description
I	Hematoma	Mild contusion without duct injury
	Laceration	Superficial laceration without duct injury

II	Hematoma	Major contusion without duct injury
	Laceration	Major laceration without duct injury or tissue loss

III	Laceration	Distal transaction or parenchymal injury with duct injury

IV	Laceration	Proximal transaction or parenchymal injury involving ampulla

V	Laceration	Massive disruption of pancreatic head

Pancreas-specific complications (PSCs) were defined as pancreatic pseudocyst, fistula and/or intra-abdominal abscess. A time interval greater than 48 hours between injury and surgery was regarded as delayed operation.

We compared the ISS and length of hospital and ICU stay of patients with PSC (PSC group) and without PSC (non-PSC group) using the Wilcoxon rank sum test. Statistical analyses were performed with two-sided significance level of 0.05 using the SPSS software package (SPSS 12.0)

## Results

### Study population

Nineteen patients with documented pancreatic trauma included fourteen male patients and five female patients, with an average age of 40.6 ± 21.4 years (range, 11 to 77 years). There were six deaths in the study population (mortality rate 31.6%), including three patients who died from severe brain injuries and three who died from torso trauma. Sixteen cases resulted from traffic accidents (8 motor vehicle, 5 motorcycle, 2 bicycle and 1 pedestrian traffic accident), two from violence and one from a fall.

### Radiological and surgical findings

Torso CT was performed in all patients and laparotomy in 14 patients. Fifteen CTs were positive for pancreatic injury and four were negative. The sensitivity of CT in detecting pancreatic trauma was 78.9% (95% confidence interval: 54%-94%). Ten deep lesions and 5 superficial lesions were identified on CT imaging. All patient with a deep lesion needed surgical intervention. Eight lesions with pancreatic duct injuries (PD+ injury) and two lesions without pancreatic duct injury (PD- injury) were discovered in laparotomy. Five patients with superficial lesions underwent non-operative management. Of the four patients with CTs which did not reveal pancreatic lesions, two PD+ and two PD- injuries were recognized during surgery. Three of these patients underwent laparotomy for hemodynamic instability and hemoperitoneum found on CT imagines. Another patient received surgery for physical examination found positive peritoneal signs. No magnetic resonance cholangiopancreatography (MRCP) and only one endoscopic retrograde cholangiopancreatography (ERCP) was conducted on a patient receiving conservative treatment and the ERCP revealed negative for pancreatic duct injury.

Most patients had more than one associated organ injury; only 5 (26.3%) had isolated pancreatic trauma. The most common injury organs were to the brain, liver, lung and spleen (Table 2).

**Table 2:** Demographic data and associated organ injuries

Age		40.6 ± 21.4
Male		74% (14/19)

ISS		36.9 ± 27.6

Trauma mechanisms	Fall	5% (1/19)

	Violence	11% (2/19)

	Traffic accident	84% (16/19)

Patients with isolated pancreatic injury		26% (5/19)
Patients with injury to the pancreas and other organs		74% (14/19)
**Associated organ injuries**		
Brain		37% (7/19)
Liver		32% (6/19)
Lung		32% (6/19)
Spleen		26% (5/19)
Bowel		21% (4/19)
Long bone		21% (4/19)
Aorta		11% (2/19)
Spine		11% (2/19)
Kidney		5% (1/19)

ISS: Injury Severity Score

### Complications

**Figure 1:**
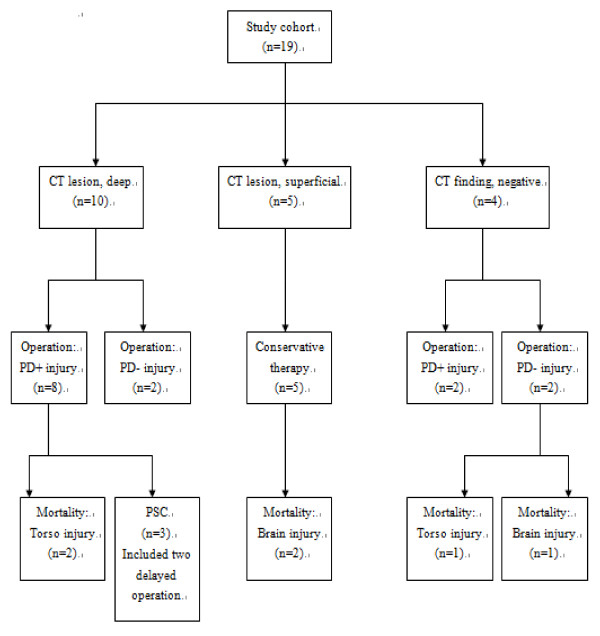
**The CT findings, operative results, mortality and complications of the study population**.

**Figure 2:**
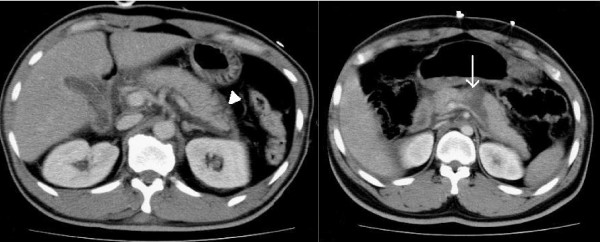
**The left picture is a patient with superficial pancreatic lesion and the right picture is a patient with deep pancreatic lesion**. The arrow head shows pancreatic swelling with focal fluid accumulation around the pancreas. The arrow shows a deep laceration transected the pancreas.

There were three PSCs, involving three patients. Twelve patients who were operated on soon after admission had one PSC and the two patients who underwent delayed operation both had PSCs. Comparing the PSC and non-PSC groups, the PSC group had longer hospital stays (51.3 and 17.4, respectively), with a trend to longer ICU stays (9.7 and 3.6, respectively), but had a tendency toward lower ISS (16.7 and 40.8, respectively). Figure 1 outlines the CT findings, operative results, mortality and complications of the study population. Figure 2 demonstrates the deep and superficial pancreatic lesions.

## Discussion

In our study population, although there was a high mortality rate, there were no deaths directly related to the pancreatic trauma. A study by Patton et al. likewise found a low mortality rate (1.6%)[12]. However, pancreatic trauma often results in prolonged morbidity [2,13,14]. We found that the ISS of the PSC group tended to lower than the non-PSC group; nevertheless, the PSC group had extended hospital stays with a trend to longer ICU stays. Regardless of the severity of trauma, PSCs caused prolonged morbidity in patients with pancreatic trauma.

Non-operative management is the accepted treatment of liver, spleen and kidney injuries in patients with trauma to the torso [15,16]. However, the role of conservative therapy in pancreatic trauma is still debated. Holmes et al. found that the failure rate of non-operative treatment for pancreatic trauma was much higher than for other abdominal solid organs [17]. It is generally accepted that surgery is not imperative in patients with low grade pancreatic trauma (grade I or II contusions or lacerations without pancreatic duct injury) [18]. Unfortunately, the integrity of the pancreatic duct is difficult to determinate. Both ERCP and MRCP can accurately evaluate the pancreatic duct, but as first-line diagnostic tools in trauma patients these modalities are controversial [10].

CT is still the most useful tool to evaluate patients with torso trauma. We found CT had an 78.9% sensitivity in diagnosing pancreatic trauma, and the extent of pancreatic injury found in the scans correlated with surgical findings. All deep lacerations and hematomas required surgery. Patients with deep pancreatic injuries who underwent delayed procedures had a 100% incidence of PSC. In addition, every superficial lesion caused by blunt trauma was successfully treated non-operatively. A study by Phelan et al. had reported the 16- or 64-mlutidetector CT had high specificity in detection of pancreatic duct injury, however, most radiographic reports of the CT scan in our institution did not mentioned the integrity of pancreatic duct [9]. Therefore, we used the depth of the pancreatic injury instead the direct description of pancreatic duct to determine the violation of pancreatic duct. Wong et al. suggested that a CT finding of a lesion of more than 50% of the thickness of the pancreas indicated likely disruption of the pancreatic duct [11]. This opinion is in line with our findings. Pancreatic trauma is a condition that potentially requires operative treatment, and the results of CT imaging could help decide further management.

The majority of patients with pancreatic trauma in our study had associated organ injuries. This makes establishing the diagnosis of pancreatic injury more difficult. Nevertheless, CT successfully diagnosed most pancreatic trauma and identified other associated organ injuries. Despite these advantages, a normal CT cannot exclude pancreatic injury. Typical trauma mechanisms (steering wheel injury in motor vehicle accidents and handlebar injury in pediatric bicycle accidents) [7,14]; repeated physical examinations and further imaging studies (ERCP, MRCP or follow-up CT) help identify occult pancreatic trauma.

## Limitations

The overall case number is small and this limits subgroup analysis.

The study design has all the inherent constraints of retrospective studies.

## Conclusions

The majority of cases of pancreatic trauma had associated organ injuries. Four-slice helical CT identified most pancreatic trauma and provided practical therapeutic guide. However, CT missed a small portion of pancreatic traumas. Patients with superficial pancreatic injuries were candidates for non-operative therapy while deep pancreatic lesions revealed on CT required surgery. Delayed operation resulted in complications and was associated with prolonged hospital stays.

## Competing interests

The authors declare that they have no competing interests.

## Authors' contributions

WJL conceived the study and drafted the manuscript. NPF collected and managed the data. HJL supervised the conduct of the trial and contributed to its revision. YCH assisted the conduct of the trial. KTC designed the trial and analyzed the data.

All authors have read and approved the final manuscript
